# Assessing Quality of Life in PACS1 Syndrome Using the KidsLife Scale from Mothers’ and Fathers’ Perspectives

**DOI:** 10.3390/bs16020250

**Published:** 2026-02-09

**Authors:** Julia del Rincón, Laura Trujillano, Cristina Lucia-Campos, Isabel Xiang, Ana Latorre-Pellicer, Beatriz Puisac, María Arnedo, Marta Gil-Salvador, Laura Acero, Pilar Pamplona, Ariadna Ayerza-Casas, Feliciano J. Ramos, Juan Pié

**Affiliations:** 1 Department of Pharmacology and Physiology, Unit of Clinical Genetics and Functional Genomics, School of Medicine, University of Zaragoza, CIBERER-GCV02 and IIS-Aragon, 50009 Zaragoza, Spain; 2 Clinical and Molecular Genetics Area, Sant Joan de Deu Children’s Hospital, 08950 Barcelona, Spain; 3 Clinical and Molecular Genetics Area, Vall d’Hebron Hospital, Medicine Genetics Group, Vall d’Hebron Research Institute (VHIR), 08035 Barcelona, Spain; 4 Unit of Neurophysiology, University Hospital Lozano Blesa, 50009 Zaragoza, Spain; 5 Unit of Paediatric Cardiology, Service of Paediatrics, Hospital Universitario Miguel Servet, 50009 Zaragoza, Spain; 6 Unit of Clinical Genetics, Service of Paediatrics, University Hospital “Lozano Blesa”, School of Medicine, University of Zaragoza, CIBERER-GCV02 and IIS-Aragon, 50009 Zaragoza, Spain; framos@unizar.es

**Keywords:** PACS1 syndrome, quality of life, intellectual disability, rare diseases, KidsLife scale, caregiver burden

## Abstract

PACS1 Syndrome is an ultra-rare neurodevelopmental disorder characterized by intellectual disability, behavioral disturbances, and multisystem involvement. While clinical knowledge is growing, its impact on quality of life (QoL) has not been systematically evaluated, and it is critical to understand the lived experience and psychosocial well-being of these individuals beyond strictly medical outcomes. This study aimed to assess QoL in individuals aged 4–21 years with PACS1 Syndrome using the validated KidsLife scale, proxy-reported by primary caregivers, given the intellectual disabilities and communicative limitations of this population. Twenty-one participants from Spain and other countries were recruited through the Spanish PACS1 Association, and 39 questionnaires from mothers and fathers were analyzed. The KidsLife scale provides standardized scores across eight QoL domains and a global QoL index (QoLI). The mean QoLI was 48.1 ± 28.3, slightly below the median for individuals with intellectual disability, but higher than other neurodevelopmental disorders such as Cornelia de Lange Syndrome. The findings revealed a pattern: while domains related to social inclusion, rights, and physical and material well-being were relatively preserved, reflecting adequate care and access to resources, the most significant compromises were observed in autonomy-related domains, specifically self-determination, interpersonal relationships, and personal development. Most individuals showed a high degree of dependency, and those with greater dependency exhibited lower QoL scores. This situation led more than half of families to reduce their working hours, with caregiving responsibilities disproportionately falling on mothers. Although no statistically significant differences were found between parental ratings, mothers tended to report higher QoL. These findings reflect the substantial functional impact of PACS1 Syndrome and emphasize the need for multidisciplinary support to improve autonomy, social participation, and overall well-being.

## 1. Introduction

Schuurs-Hoeijmakers Syndrome (SHS) (MIM #615009), also known as PACS1 Neurodevelopmental Disorder (PACS1-NDD) or PACS1, is an ultra-rare genetic disease (with only about 100 patients reported to date (Arnedo et al., 2022)) caused by pathogenic variants in the *PACS1* gene. The condition was first described in 2012 by Schuurs-Hoeijmakers and colleagues (Schuurs-Hoeijmakers et al., 2012).

Clinically, the syndrome is characterized by distinctive craniofacial features, global developmental delay with intellectual disability, and variably associated ocular, urogenital, or cardiac anomalies. Patients frequently present with hypotonia, seizures with highly variable onset—including during the first days of life—and feeding difficulties (del Rincón et al., 2025; Latorre-Pellicer et al., 2023; Lusk et al., 2020; Schuurs-Hoeijmakers et al., 2016; Tenorio-Castaño et al., 2021). Cognitive and behavioral functioning is among the most affected domains: intellectual disability is universal and ranges from mild to profound, with language development consistently delayed and often more impaired than motor skills, and sometimes absent (Schuurs-Hoeijmakers et al., 2016; Van Nuland et al., 2021). Motor abilities are also commonly affected; while many individuals achieve independent walking, others experience ataxia or gait instability requiring mobility aids, and some show motor decline over time, which may indicate a tendency toward neurodegeneration (Arnedo et al., 2022; Lusk et al., 2020; Schuurs-Hoeijmakers et al., 2016; Van Nuland et al., 2021). Although individuals with PACS1 Syndrome are often described as friendly, behavioral difficulties—including tantrums, self-injury, and aggression—are frequent, and autism spectrum disorder is reported in a substantial subset of patients (Lusk et al., 2020; Stern et al., 2017; Van Nuland et al., 2021).

Although experience with other neurodevelopmental syndromes shows that behavioral impairment and systemic involvement (referring to the multisystemic manifestations such as cardiac, renal, and neurological comorbidities) can negatively affect a patient’s quality of life (QoL), systematic studies evaluating QoL in individuals with PACS Syndrome are still lacking.

Furthermore, this study aims to address the less visible dimensions of living with rare neurodevelopmental disorders. Despite advances in clinical and genetic characterization, children and young people with rare conditions often remain underrepresented in research focusing on participation, inclusion, and accessibility (Sáiz-Manzanares et al., 2024). Emphasizing this imbalance allows us to position this work as a response to the tendency to privilege clinical outcomes over everyday lived experiences.

QoL can be defined as a multidimensional state of personal well-being that encompasses cultural dimensions as well as subjective and objective aspects, and is influenced by individual and environmental factors. Several validated questionnaires are available for the study of QoL in individuals with intellectual disabilities (Bullinger et al., 2002; Dickinson et al., 2007; Varni & Limbers, 2008). One of the most robust is the KidsLife scale (Gómez et al., 2016).

The scale provides broad coverage across eight fundamental domains of QoL: social inclusion, self-determination, emotional well-being, physical well-being, material well-being, rights, personal development, and interpersonal relations. Each domain is assessed through 12 questions with four possible responses, ranging from 1 to 4 (never, sometimes, often, and always, respectively).

This multidimensional instrument is specifically designed and validated for assessing individuals with intellectual disabilities from the perspective of their parents or primary caregivers (proxy-report). Quality of life assessment via caregivers is a standard and theoretically grounded approach in populations with severe intellectual disability and limited communicative abilities (Wang et al., 2006), as well as a widely used approach in contexts where self-report is not feasible.

The selection of the KidsLife scale for assessing QoL was based on several factors:

First, it is a universally validated tool for assessing individuals with intellectual disabilities from the perspective of their parents or caregivers, demonstrating adequate validity and reliability for the age range of our cohort (children and young individuals). Its reliability is supported by a large normative sample of 1060 individuals with intellectual disabilities aged 4 to 21 years, which allowed to calculate percentiles for standardizing the results (Gómez et al., 2014; Gómez et al., 2016).

Second, the scale is validated in both Spanish and English, allowing a wider range of participants to be accurately assessed (Gómez et al., 2014; Gómez et al., 2016).

Finally, it has been applied with satisfactory results in diverse genetic syndromes with different molecular bases, as demonstrated in previous studies involving Down Syndrome (Trisomy 21, DS) (Lee et al., 2021), a chromosomal condition associated with mild to moderate intellectual disability (Antonarakis et al., 2020); Williams Syndrome (WS) (Moraleda Sepúlveda & López Resa, 2021), caused by a 7q11.23 deletion and characterized by moderate intellectual disability with relatively stronger language skills (Kozel et al., 2022); and Cornelia de Lange Syndrome (CdLS) (Trujillano et al., 2024), a monogenic disorder—similar to PACS1 Syndrome—but generally presenting with more severe intellectual disability, marked behavioral challenges, and multisystemic involvement (Ascaso et al., 2024).

As PACS1 Syndrome is a chronic condition, with no curative treatment and multisystemic involvement, symptomatic management remains the main strategy for affected individuals. It is necessary to achieve a comprehensive understanding of QoL in these patients to implement targeted interventions by healthcare professionals, educators, and social services that address the specific challenges faced by the patients and their families, as requested by family associations. This study assesses, for the first time, the QoL in individuals with the ultra-rare disease PACS1 Syndrome.

## 2. Materials and Methods

### 2.1. Participants

The study involved a sample of 21 individuals with a molecularly confirmed diagnosis of PACS1 Syndrome (9 females and 12 males, see Table 1. The participants’ ages ranged from 4 to 21 years (median age 11 ± 5.8 years). Due to the ultra-rare nature of the syndrome—with only approximately 100 cases reported worldwide—patient recruitment was conducted over an extended four-year period (2021–2025) to ensure a representative cohort. The sample was international and heterogeneous, including participants from Spain (52.4%), other European countries (Portugal, United Kingdom, Belgium, The Netherlands, Ukraine), as well as Australia, the United States, and Latin America (Mexico, Argentina). All participants were either members of a PACS1 Familiar Association or had established contact with the Spanish PACS1 Association seeking support or collaboration.

Inclusion criteria were: (a) a molecular diagnosis of PACS1 Syndrome, (b) age between 4 and 21 years, (c) having known the participant for at least six months, and (d) having had the opportunity to observe the participant over extended periods in different situations.

### 2.2. Instrument

As detailed in the Introduction, the instrument chosen for this study was the KidsLife scale (Gómez et al., 2016), which is based on the multidimensional model of quality of life proposed by Schalock and Verdugo (Schalock et al., 2010). The Spanish version is freely available for download online via the University of Salamanca. (https://uvadoc.uva.es/bitstream/handle/10324/46984/Escala-KidsLife.pdf?sequence=2&isAllowed=y, accessed 7 February 2026). This scale was specifically selected due to its validation for proxy-reporting by caregivers.

The scale evaluates eight domains: social inclusion, self-determination, emotional well-being, physical well-being, material well-being, rights, personal development, and interpersonal relations. It consists of 96 items (12 per domain) rated on a four-point Likert scale (1 = never to 4 = always). For the analysis, the direct scores obtained in each domain were adjusted to standardized scores and their corresponding percentiles based on the participant’s age. These scores were derived from a normative sample of 1060 individuals with intellectual disabilities aged 4 to 21 years. The total standard score (Overall QoL) was calculated by summing the domain-specific standardized scores and transforming them into a QoL Index (QoLI), which follows a normal distribution (mean = 100; SD = 15). This transformation allows for a precise comparison of the individuals’ well-being regardless of their specific age group.

### 2.3. Procedure

Participants were recruited through the Spanish PACS1 Association during annual Scientific and Family meetings. The recruitment and data collection followed a sequential protocol, beginning with an initial contact where families were informed about the project during meetings or via the Association’s network. Subsequently, informed consent and clinical/genetic information were gathered using standardized forms. Parents completed the questionnaires either in person during the annual meetings (lasting 20–30 min) or via an online questionnaire for families who joined later. To ensure a comprehensive perspective, both mothers (*n* = 21) and fathers (*n* = 18) were encouraged to complete the survey independently. The questionnaire was provided in Spanish to families whose first language is Spanish, and in English to all other participants. All the non-Spanish-speaking families were fluent in English and able to understand all questions. The same group of clinical geneticists trained in the KidsLife tool was available to address questions both in person and remotely to ensure the accuracy of the responses.

### 2.4. Ethical Considerations

The study was conducted in accordance with the Declaration of Helsinki and the protocol was approved by the Ethics Committee of Clinical Research from the Government of Aragón (Spain) (CEICA, Protocol No. PI19/247 and PI24/288). All legal guardians provided written informed consent prior to participation. To ensure confidentiality and anonymity, all participant data were pseudonymized using unique identification codes, and data storage complied with current personal data protection regulations.

### 2.5. Statistical Analysis

Statistical analysis was performed using IBM SPSS Statistics (Version 29). Given the small sample size (*n* = 21) and the non-normal distribution of the data, non-parametric tests were utilized to ensure analytical rigor. The Mann–Whitney U test was employed to compare QoL scores between groups (e.g., mothers vs. fathers, and levels of dependency). Fisher’s exact test was used for categorical sociodemographic variables. Results are presented as means, standard deviations, and percentiles, with a significance threshold of *p* < 0.05.

## 3. Results

The cohort consisted of 21 participants with a confirmed diagnosis of PACS1 Syndrome. A total of 39 surveys from these participants were analyzed, 21 completed by mothers and 18 by fathers. Of the 21 participants, 13 (61.9%) belonged to families with a reduced parental work schedule, and 8 (38.1%) to families without such a reduction (Table 2).

Table 1 presents a descriptive analysis of the cohort of 21 individuals, comparing their sociodemographic and functional characteristics.

**Table 1:** Sociodemographic and functional characteristics of the cohort.

Characteristics	Total (*n* = 21)
Age [median (p25–p75)].	11 (7–18)
Gender	
Female	9 (43%)
Male	12 (57%)
Nationality	
Spanish	11 (52%)
Rest of Europe	5 (24%)
Others	5 (24%)
Single parent family	
Yes	2 (10%)
No	19 (90%)
Reduction in working hours	
Yes	13 (62%)
No	8 (38%)
Mother	10 (77%)
Father	2 (15%)
Both	1 (8%)
Recognized level of dependency (*n* = 11)	
Grade I (moderate)	2 (18%)
Grade II (severe)	3 (27%)
Grade III (high dependency)	6 (55%)
Type of schooling	
Ordinary	2 (9%)
Special	13 (62%)
Combined	6 (29%)
Level of needs	
Mother [median (p25–p75)].	3 (2–4)
Parent [median (p25–p75)	4 (3–4)

Note: Age and level of needs are expressed as medians. All other variables are expressed as absolute frequencies and percentages. Level of needs: 2 = intermittent; 3 = extensive; 4 = generalized. Only Spanish participants (*n* = 11) were considered for the level of dependence.

**Table 2:** Reduction in parental work hours in families of children with PACS1 Syndrome.

Work Time Reduction Status	Frequency (%) *n* = 21	*p* Value
None	8 (38.1%)	
Yes	13 (61.9%)	
Mother	10 (76.9%)	
Father	2 (15.4%)	
Both	1 (7.7%)	<0.005

Note: Values are expressed as absolute frequencies and percentages. The *p*-value represents the comparison among families with reduced work hours according to whether the mother, the father, or both parents reduced their working hours, using Fisher’s exact test.

The median age of participants was 11 ± 5.8 years, lower in the group with reduced working hours (9 ± 5.3) compared to the group without reduced working hours (15.5 ± 6.8).

Most participants were from Spain (52.4%), with the remaining individuals originating from various countries, including Portugal, the United Kingdom, Belgium, The Netherlands, Ukraine, Australia, Mexico, the United States, and Argentina. Reduced working hours were more frequent among non-Spanish families (61.5% vs. 38.5%). Two families (9.5%) were single-parent households, one with reduced working hours and the other without.

Regarding the degree of dependency and support needs, most participants (57.1%) were classified as having grade III dependency and extensive support needs (42.9%). Reduced parental working hours were more common in these two groups (69.2% and 46.2%, respectively).

Finally, most participants attended special education centers (61.9%), the majority of whom belonged to families with reduced working hours (69.2%). In contrast, none of the participants attending mainstream education centers (25%) came from families with reduced parental working hours.

### 3.1. Quality of Life According to Parents’ Perception

**Figure 1:**
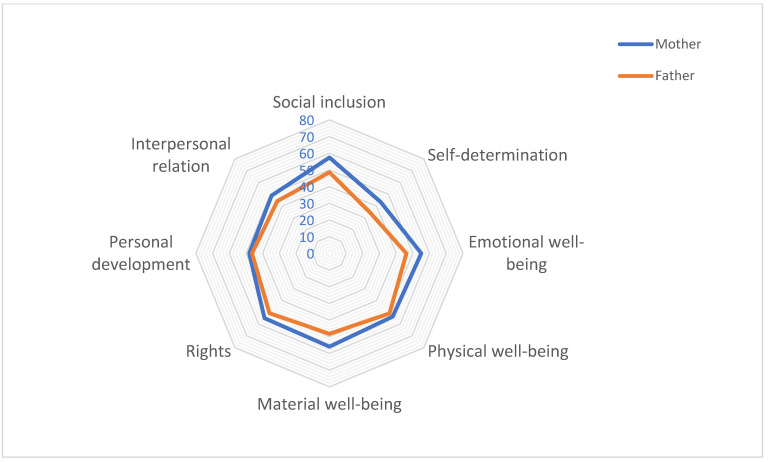
Radar chart of quality-of-life domains comparing mothers’ and fathers’ perceptions.

Table 3 and Figure 1 present the evaluation of quality of life (QoL) domains as reported by mothers and fathers of children with PACS1 Syndrome.

The highest-rated domain was social inclusion (53.4 ± 32.3), followed closely by rights (53.1 ± 31.3), physical well-being (52.4 ± 29.1), material well-being (52.3 ± 27.0), and emotional well-being (50.9 ± 30.9). The lowest-scoring domains were self-determination (39.4 ± 28.5), interpersonal relationships (46.7 ± 29.2), and personal development (47.3 ± 33.0). The Overall QoLI was 48.1 ± 28.3.

When stratifying data by parent perception, mothers gave slightly higher ratings across all domains. However, none of the differences reached statistical significance (all *p*-values > 0.05). In the domain of emotional well-being, mothers reported a mean of 55.0 ± 28.3) while fathers reported 46.1 ± 33.9 (*p* = 0.335). Similar non-significant differences were found in social inclusion (mothers 57.5± 35.3 vs. fathers 48.6 ± 28.5; *p* = 0.364), self-determination (mothers 43.5 ± 31.3 vs. fathers 34.5 ± 24.8; *p* = 0.443), and material well-being (mothers 55.9 ± 25.6 vs. fathers 48.2 ± 28.7; *p* = 0.443).

Other domains, including physical well-being, rights, personal development, and interpersonal relationships, showed similar scores between mothers and fathers, with no statistically significant differences.

The Overall QoLI was also higher in mothers (51.4 ± 27.4) than in fathers (44.3 ± 29.7), although this difference was not statistically significant (*p* = 0.443).

### 3.2. Quality of Life by Recognized Dependency Level

Table 4 presents the values of various QoL domains according to the level of dependency (Grade I + II vs. Grade III). Only Spanish participants (*n* = 11) were included, following Law 39/2006 as outlined in Official State Bulletin No. 299. Overall, individuals with lower dependency level (Grades I and II) showed higher scores across most evaluated domains compared to those with severe dependency (Grade III).

Numerical differences were observed in the domain of self-determination, with individuals in the lower dependency group having a mean of 51.3 ± 20.3, compared to 23.2 ± 25.0 in the higher dependency group (*p* = 0.052).

In the rights domain, the scores were 68.6 ± 26.0 in Grades I and II versus 39.8 ± 31.0 in Grade III, although this did not reach statistical significance (*p* = 0.126).

The QoLI was higher in the lower dependency group (57.0 ± 21.0 vs. 43.4 ± 32.3; *p* = 0.537). This pattern was similarly observed in other domains, such as physical well-being, material well-being, and social inclusion, although none of the differences reached statistical significance (*p* > 0.05).

Conversely, the only domain in which the higher dependency group had a slightly higher average score was emotional well-being (54.5 ± 32.4 vs. 48.8 ± 19.3), which was also not statistically significant (*p* = 0.792).

## 4. Discussion

PACS1 Syndrome is a rare disease, recently characterized from a clinical perspective, presenting a wide spectrum of neurocognitive, behavioral, and physical manifestations. Most studies to date have focused on its genetic and clinical aspects, while its impact on the quality of life (QoL) of affected individuals has been scarcely explored. Given the lack of curative treatment and the complexity of its clinical presentation, assessing the overall well-being of these individuals is essential.

All reported cases exhibited intellectual disability as part of the neurodevelopmental phenotype. A high frequency of language delay, autism spectrum disorder, behavioral issues, seizures, and other physical problems was also observed, with similar prevalence to those reported in previous studies (Pagano et al., 2025; Schuurs-Hoeijmakers et al., 2016; Seto et al., 2021; Tenorio-Castaño et al., 2021).

From a sociodemographic perspective, the impact on family life was substantial. More than half of the families had to reduce the working hours of at least one parent, predominantly mothers. This finding is consistent with previous studies (Boettcher et al., 2021; Fernández-Ávalos et al., 2020; Walkowiak & Domaradzki, 2025) that highlight gender inequalities in caregiving roles in rare diseases and underscore the need for policies that support work–life balance and institutional assistance.

Functionally, most individuals exhibited a high degree of dependency, requiring significant support in daily life. While the quantitative differences in QoL scores between parents did not reach statistical significance (*p* > 0.05), a qualitative distinction was observed in their perceptions: fathers tended to perceive these demands as more burdensome. This possibly reflects differences in how each parent interprets the syndrome’s impact on family life.

Regarding the choice of the assessment instrument, it is pertinent to address the high prevalence of Autism Spectrum Disorder (ASD) within the PACS1 population (25% to 30% of individuals) (Lusk et al., 2020). Although a specific version—the KidsLife-ASD—was developed to increase sensitivity to the particular needs of individuals with autism (Gómez et al., 2020), the standard KidsLife scale was selected for this study for several reasons. First, while ASD is a common feature of the PACS1 behavioral phenotype, it is not a universal diagnosis across our entire sample; thus, the general version ensured a more inclusive evaluation of all participants. Second, PACS1 is a complex, multisystemic syndrome with significant organic involvement. The standard KidsLife scale provides a holistic assessment of dimensions such as Physical and Material Well-being, which are essential to capture the impact of these systemic medical challenges on the patient’s overall quality of life. Nonetheless, we recognize that future research focusing specifically on the behavioral nuances of PACS1 might benefit from the KidsLife-ASD to further explore social-communication and sensory-processing impacts.

QoL evaluation using the KidsLife scale revealed considerable variability across individuals and domains. The cohort’s QoLI was 48.1 ± 28.3, slightly higher than that reported for Cornelia de Lange Syndrome (CdLS, 45.3 ± 31.1) (Trujillano et al., 2024), but still below the median observed in individuals with other intellectual disabilities and lower than that reported Down Syndrome (70–71st percentile) (Lee et al., 2021). Although these comparisons with other syndromes are strictly descriptive and do not imply statistical equivalence or direct inferential comparison, the proximity of the scores to CdLS results underscores the severe functional impact of PACS1.

The highest scores were observed in domains such as social inclusion (53.4 ± 32.3), rights (53.1 ± 31.3), and physical, material, and emotional well-being (52.4 ± 29.1, 52.3 ± 27.0, and 50.9 ± 30.9, respectively). From a statistical perspective, these scores did not represent a significant departure from the normative median of the intellectual disability population (*p* > 0.05). Nevertheless, they carry substantial clinical weight by highlighting specific dimensions of relative strength where the syndrome’s impact is less severe. These results suggest that, in both Spanish and international families, despite clinical severity of PACS1 Syndrome, many families perceive that their children’s rights are respected, that they live in a supportive family environment, and that their basic needs are adequately met through consistent, high-quality care and access to essential resources and services. Therefore, the detected decrease in QoL is likely attributable to their underlying health problems rather than to deficiencies in care. Nevertheless, the large standard deviations indicate considerable variability, likely influenced by socioeconomic context or individual family experiences.

Conversely, the lowest scores were noted in the domains of self-determination, interpersonal relationships, and personal development areas, reflecting significant limitations in autonomy, social skills, and daily life competencies. It is essential to interpret these low scores in self-determination not merely as an inherent limitation of the individual with PACS1 Syndrome, but as a result of the structural, educational, and social constraints they face. Often, individuals with high support needs are provided with fewer opportunities to exercise choice and control over their own lives, meaning these results may reflect a lack of environmental affordances rather than a lack of potential for agency. By framing autonomy as a shared responsibility between the individual and their environment, we can better address the systemic invisibility of rare neurodevelopmental disorders in educational and social research. This highlights the need for specific interventions, such as individualized educational programs and evidence-based behavioral supports that prioritize empowerment and social policies that remove barriers to participation. In this regard, evidence-based approaches, such as Applied Behavior Analysis (ABA), among others, have been shown to be effective in addressing behavioral challenges and promoting skill acquisition in children with neurodevelopmental disorders (Benavidez et al., 2024; Laureano et al., 2023; Lusk et al., 2020).

These results differ from the ones found in Down Syndrome, where the highest-scoring domains were social inclusion (84th percentile), self-determination (75th percentile), and material well-being (63rd percentile), while the lowest-scoring domains were emotional well-being (37th percentile), physical well-being (50th percentile), and interpersonal relations (50th percentile). This pattern may be explained by the relatively strong cognitive and social functioning observed in individuals with Down Syndrome (Lee et al., 2021).

However, the QoL results in individuals with PACS1 Syndrome are consistent with those previously reported in CdLS (Trujillano et al., 2024), where the domains with the highest percentile scores were material well-being (59.7 ± 28.2), rights (55.8 ± 30.1), and interpersonal relations (44.9 ± 32.4), as well as in Williams Syndrome (Moraleda Sepúlveda & López Resa, 2021) (WS; material and physical well-being, and rights). The lowest percentile scores were observed in self-determination (41 ± 35.1), personal development (42.8 ± 30.4), and physical well-being (43.8 ± 30.8) in CdLS, a pattern also observed in WS. These findings reinforce the idea that these individuals receive adequate care and that their basic needs are being met. Moreover, particularly in CdLS and, to a lesser extent, in PACS1 Syndrome, it is plausible that the association with greater intellectual disability and chronic physical comorbidities imposes intrinsic limits. Thus, even when support systems are optimized and needs are fully addressed, the inherent burden of the clinical severity of the condition may constrain the perceived QoL, suggesting that high-quality care cannot entirely offset the impact of the condition, regardless of the efficacy of the external supports provided.

Several studies have shown that psychosocial factors, such as limited access to services, communication difficulties with healthcare professionals, and feelings of isolation or frustration, significantly affect caregivers’ QoL (Boettcher et al., 2021; Walkowiak & Domaradzki, 2025). In some cases, this impact may be greater than that caused by the severity of the disease itself. These conditions, by affecting the emotional and functional well-being of parents, may also influence their perception of their child’s QoL. This connection underscores that the assessment of a child’s QoL in ultra-rare diseases like PACS1 is inseparable from the family’s lived experience. In this context, support programs and community networks play a key protective role. Notably, rare disease associations such as the Spanish PACS1 Association provide crucial support to both Spanish and international families, helping to improve overall well-being.

When comparing QoL perception by parent gender, no statistically significant differences were found, although mothers tended to rate all domains slightly higher, particularly in emotional well-being, social inclusion, and self-determination. This slight divergence might reflect the non-neutral nature of proxy-reporting, potentially influenced by the greater maternal involvement in caregiving, a trend previously noted in families dealing with rare diseases (Boettcher et al., 2021; Fernández-Ávalos et al., 2020; Walkowiak & Domaradzki, 2025).

In addition, regarding the recognized dependency level, although no statistically significant differences were observed (*p* > 0.05), scores were generally higher in individuals with lower dependency, especially in the domains of self-determination and rights. The most notable difference was in self-determination (*p* = 0.052), consistent with prior findings in WS, where significant QoL differences were reported depending on the level of dependency (Moraleda Sepúlveda & López Resa, 2021). This underscores autonomy as a key factor in QoL evaluation in neurodevelopmental disorders, with dependency level playing an important role in understanding these variations.

In contrast, participants with higher dependency scored higher in emotional well-being, a finding similar to that reported in the WS study (Moraleda Sepúlveda & López Resa, 2021), though not statistically significant. This may be related to better psychological adaptation, consistent family support, and a person-centered care model that helps maintain emotional stability despite high dependency. These findings support the utility of the dependency grading system as a functional indicator for QoL assessment in this population.

The main limitation of this study is the sample size. Including only 21 individuals restricts the statistical power of the analysis. However, PACS1 Syndrome is an ultra-rare condition, with approximately one hundred cases reported worldwide. Furthermore, the cohort comprises, in addition to the Spanish population, individuals of different nationalities linked to the Spanish PACS1 Syndrome Association, which may influence the results due to sociodemographic differences. Additionally, the cross-sectional design of the study and the potential variability of QoL across different developmental stages are acknowledged as limitations. These factors emphasize the importance of conducting future longitudinal research to provide a more dynamic understanding of how quality of life and support needs evolve over time in this population. Another limitation is the use of the KidsLife scale, which is completed by the patient’s parents or caregivers rather than the affected individuals themselves. Nevertheless, it should be noted that, in most cases, the affected individuals were unable to complete this type of survey independently.

## 5. Conclusions

In conclusion, this study represents the first comprehensive assessment of the QoL in individuals affected by PACS1 Syndrome. Individuals with this disorder exhibit slightly higher QoL compared to those with other intellectual disabilities, such as CdLS, which may be attributed to less severe multisystemic involvement, yet their QoL remains below the median observed in individuals with intellectual disability.

Most individuals exhibit a high degree of dependency, which leads over half of the families to reorganize their working schedules by reducing working hours. Additionally, caregiving responsibilities are unequally distributed, with mothers bearing the greater burden. This underscores a gender inequity that should be addressed through public policies promoting work–life balance and institutional support. Although no statistically significant differences were found in parental perceptions, fathers tended to assign lower ratings across all QoL domains.

Evaluation of QoL using the KidsLife scale showed high scores in domains such as social inclusion, rights, and physical and material well-being. Conversely, lower scores in self-determination and interpersonal relationships reflect the significant structural and social barriers to autonomy faced by these individuals. The data also suggest that greater dependency is associated with lower overall QoL.

These findings have direct implications for clinical practice and educational planning. Medical management must go beyond symptom control to address the functional and social challenges that impact well-being, while individualized educational programs should prioritize autonomy and social skills over strictly academic goals. Furthermore, the substantial impact on family life calls for the strengthening of support services and community networks to alleviate caregiver burden and promote gender equity in care.

Ultimately, the complexity of PACS1 Syndrome requires multidisciplinary approaches that integrate clinical care with psychosocial support and evidence-based interventions to enhance the quality of life for both affected individuals and their families. By providing a baseline of QoL in this ultra-rare population, this study aims to lay the groundwork for future comparative research with other neurodevelopmental disorders and for longitudinal studies that track how QoL and support needs evolve across different developmental stages.

## Figures and Tables

**Table 3:** Comparison of quality of life domains reported by mothers and fathers of children with PACS Syndrome.

Domains	Total (*n* = 39)	Mother (*n* = 21)	Father (*n* = 18)	*p*-Value
**SOCIAL INCLUSION**	53.4 ± 32.3	57.5 ± 35.3	48.6 ± 28.5	0.364
**SELF-DETERMINATION**	39.4 ± 28.5	43.5 ± 31.3	34.5 ± 24.8	0.443
**EMOTIONAL WELL-BEING**	50.9 ± 30.9	55.0 ± 28.3	46.1 ± 33.9	0.335
**PHYSICAL WELL-BEING**	52.4 ± 29.1	53.6 ± 28.3	50.9 ± 30.9	0.770
**MATERIAL WELL-BEING**	52.3 ± 27.0	55.9 ± 25.6	48.2 ± 28.7	0.443
**RIGHTS**	53.1 ± 31.3	55.0 ± 29.7	50.8 ± 33.8	0.587
**PERSONAL DEVELOPMENT**	47.3 ± 33.0	48.1 ± 34.0	46.4 ± 32.6	0.606
**INTERPERSONAL RELATIONSHIPS**	46.7 ± 29.2	49.0 ± 27.9	44.2 ± 31.2	0.646
**QUALITY OF LIFE INDEX (QLI)**	48.1 ± 28.3	51.4 ± 27.4	44.3 ± 29.7	0.443

Note: Values are expressed as mean ± standard deviation of percentiles for each QoL domain and for the overall QoL index. Comparisons between mothers (*n* = 21) and fathers (*n* = 18) were performed using the Mann–Whitney U test. Two participants had only maternal responses and one father did not complete the questionnaire.

**Table 4:** Comparison of quality of life domains according to the level of recognized dependence.

Domains	Total (*n* = 11)	Grade I + II (*n* = 5)	Grade III (*n* = 6)	*p*-Value
**SOCIAL INCLUSION**	57.3 ± 28.2	61.6 ± 28.7	53.8 ± 20.1	0.792
**SELF-DETERMINATION**	36.0 ± 26.3	51.3 ± 20.3	23.2 ± 25.0	0.052
**EMOTIONAL WELL-BEING**	51.9 ± 26.1	48.8 ± 19.3	54.5 ± 32.4	0.792
**PHYSICAL WELL-BEING**	51.6 ± 25.8	58.0 ± 23.5	46.3 ± 28.4	0.662
**MATERIAL WELL-BEING**	55.2 ± 24.1	61.6 ± 15.6	49.8 ± 29.8	0.662
**RIGHTS**	52.9 ± 31.3	68.6 ± 26.0	39.8 ± 31.0	0.126
**PERSONAL DEVELOPMENT**	46.6 ± 33.9	49.4 ± 34.2	44.3 ± 36.7	0.931
**INTERPERSONAL RELATIONSHIPS**	52.2 ± 28.8	53.5 ± 23.3	51.1 ± 35.0	0.792
**QUALITY OF LIFE INDEX (QLI)**	49.6 ± 27.4	57.0 ± 21.0	43.4 ± 32.3	0.537

Note: Only Spanish participants were considered, according to the level of recognized dependency. Given the small number of individuals with grade I and II dependence compared to grade III, participants were grouped into two categories: I-II (moderate to severe dependence) and III (high dependence). Values are expressed as mean ± standard deviation of the percentiles obtained for each quality of life domain, as well as for the overall QoLI. Comparisons between groups I + II (*n* = 5) and III (*n* = 6) were performed using the Mann–Whitney U test.

## Data Availability

The data presented in this study are available on request from the corresponding author due to privacy restrictions.

## Abbreviations

The following abbreviations are used in this manuscript:

ADLs	Activities of Daily Living
ASD	Autism Spectrum Disorder
CdLS	Cornelia de Lange Syndrome
DS	Down Syndrome
ID	Intellectual Disability
NDD	Neurodevelopmental Disorder
PACS1	Phosphofurin Acidic Cluster Sorting protein 1
QoL	Quality of Life
QoLI	Quality of Life Index
SD	Standard Deviation
SHS	Schuurs-Hoeijmakers Syndrome
WS	Williams Syndrome

## References

[B1-behavsci-16-00250] Antonarakis S. E., Skotko B. G., Rafii M. S., Strydom A., Pape S. E., Bianchi D. W., Sherman S. L., Reeves R. H. (2020). Down syndrome. Nature Reviews Disease Primers.

[B2-behavsci-16-00250] Arnedo M., Ascaso A., Latorre-Pellicer A., Lucia-Campos L., Gil-Salvador M., Ayerza-Casas A. (2022). Molecular basis of the Schuurs-Hoeijmakers syndrome: What we know about the gene and the PACS-1 protein and novel therapeutic approaches. International Journal of Molecular Sciences.

[B3-behavsci-16-00250] Ascaso A., Arnedo M., Puisac B., Latorre-Pellicer A., del Rincón J., Bueno-Lozano G., Pié J., Ramos F. J. (2024). Cornelia de lange spectrum. Anales de Pediatría (English Edition).

[B4-behavsci-16-00250] Benavidez H. R., Johansson M., Jones E., Rea H., Kurtz-Nelson E. C., Miles C., Whiting A., Eayrs C., Earl R., Bernier R. A., Eichler E. E., Neuhaus E. (2024). Predicting Intervention Use in Youth with Rare Variants in Autism-Associated Genes. Journal of Autism and Developmental Disorders.

[B5-behavsci-16-00250] Boettcher J., Boettcher M., Wiegand-Grefe S., Zapf H. (2021). Being the pillar for children with rare diseases—A systematic review on parental quality of life. International Journal of Environmental Research and Public Health.

[B6-behavsci-16-00250] Bullinger M., Schmidt S., Petersen C. (2002). Assessing quality of life of children with chronic health conditions and disabilities: A European approach. International Journal of Rehabilitation Research.

[B7-behavsci-16-00250] del Rincón J., Gil-Salvador M., Lucia-Campos C., Acero L., Trujillano L., Arnedo M., Pamplona P., Ayerza-Casas A., Puisac B., Ramos F. J., Pié J., Latorre-Pellicer A. (2025). AI-based facial phenotyping supports a shared molecular axis in PACS1-, PACS2-, and WDR37-related syndromes. International Journal of Molecular Sciences.

[B8-behavsci-16-00250] Dickinson H. O., Parkinson K. N., Ravens-Sieberer U., Schirripa G., Thyen U., Arnaud C., Beckung E., Fauconnier J., McManus V., Michelsen S. I., Parkes J., Colver A. F. (2007). Self-reported quality of life of 8-12-year-old children with cerebral palsy: A cross-sectional European study. The Lancet.

[B9-behavsci-16-00250] Fernández-Ávalos M. I., Pérez-Marfil M. N., Ferrer-Cascales R., Cruz-Quintana F., Clement-Carbonell V., Fernández-Alcántara M. (2020). Quality of life and concerns in parent caregivers of adult children diagnosed with intellectual disability: A qualitative study. International Journal of Environmental Research and Public Health.

[B10-behavsci-16-00250] Gómez L. E., Alcedo M., Arias B., Fontanil Y., Arias V., Monsalve A., Verdugo M. (2016). A new scale for the measurement of quality of life in children with intellectual disability. Research in Developmental Disabilities.

[B11-behavsci-16-00250] Gómez L. E., Morán M. L., Alcedo M. Á., Arias V. B., Verdugo M. (2020). Addressing quality of life of children with autism spectrum disorder and intellectual disability. Intellectual and Developmental Disabilities.

[B12-behavsci-16-00250] Gómez L. E., Verdugo M. Á., Arias B., Navas P., Schalock R. L. (2014). El constructo de calidad de vida en niños y adolescentes con discapacidades múltiples y profundas: Propuesta para su evaluación. Siglo Cero.

[B13-behavsci-16-00250] Kozel B. A., Barak B., Kim C. A., Mervis C. B., Osborne L. R., Porter M., Pober B. R. (2022). Williams syndrome. Nature Reviews Disease Primers.

[B14-behavsci-16-00250] Latorre-Pellicer A., Trujillano L., del Rincón J., Peña-Marco M., Gil-Salvador M., Lucia-Campos C., Arnedo M., Puisac B., Ramos F. J., Ayerza-Casas A., Pié J. (2023). Heart Disease Characterization and Myocardial Strain Analysis in Patients with PACS1 Neurodevelopmental Disorder. Journal of Clinical Medicine.

[B15-behavsci-16-00250] Laureano B., Fernandez N., Hagopian L. P. (2023). Efficacy of competing stimulus assessments: A summary of 35 consecutively encountered cases. Journal of Applied Behavior Analysis.

[B16-behavsci-16-00250] Lee A., Knafl G., Knafl K., Van Riper M. (2021). Quality of life in individuals with Down syndrome aged 4 to 21 years. Child: Care, Health and Development.

[B17-behavsci-16-00250] Lusk L., Smith S., Martin C., Taylor C., Chung W. (2020). PACS1 neurodevelopmental disorder synonym: Schuurs-Hoeijmakers syndrome. *GeneReviews^®^—NCBI Bookshelf*.

[B18-behavsci-16-00250] Moraleda Sepúlveda E., López Resa P. (2021). Evaluating quality of life in families with Williams Syndrome patients. Health and Quality of Life Outcomes.

[B19-behavsci-16-00250] Pagano S., Lopergolo D., De Falco A., Meossi C., Satolli S., Pasquariello R., Trovato R., Tessa A., Casalini C., Pfanner L., Astrea G., Battini R., Santorelli F. M. (2025). Expanding the clinical spectrum associated with the recurrent Arg203Trp variant in *PACS1*: An Italian cohort study. Genes.

[B20-behavsci-16-00250] Sáiz-Manzanares M. C., Casas-Cortés I., Marticorena-Sánchez R., Rodríguez-Diez J. J. (2024). Impact of Artificial Intelligence and Virtual Reality on Educational Inclusion: A Systematic Review of Technologies Supporting Students with Disabilities. Education Sciences.

[B21-behavsci-16-00250] Schalock R. L., Keith K. D., Verdugo M. Á., Gómez L. E., Kober R. (2010). Quality of life model development and use in the field of intellectual disability. Enhancing the quality of life of people with intellectual disabilities.

[B22-behavsci-16-00250] Schuurs-Hoeijmakers J. H. M., Landsverk M. L., Foulds N., Kukolich M. K., Gavrilova R. H., Greville-Heygate S., Hanson-Kahn A., Bernstein J. A., Glass J., Chitayat D., Burrow T. A., Husami A., Collins K., Wusik K., van der Aa N., Kooy F., Brown K. T., Gadzicki D., Kini U., Brunner H. G. (2016). Clinical delineation of the PACS1-related syndrome-Report on 19 patients. American Journal of Medical Genetics, Part A.

[B23-behavsci-16-00250] Schuurs-Hoeijmakers J. H. M., Oh E. C., Vissers L. E. L. M., Swinkels M. E. M., Gilissen C., Willemsen M. A., Holvoet M., Steehouwer M., Veltman J. A., De Vries B. B. A., Van Bokhoven H., De Brouwer A. P. M., Katsanis N., Devriendt K., Brunner H. G. (2012). Recurrent *de novo* mutations in *PACS1* cause defective cranial-neural-crest migration and define a recognizable intellectual-disability syndrome. American Journal of Human Genetics.

[B24-behavsci-16-00250] Seto M. T. Y., Bertoli-Avella A. M., Cheung K. W., Chan K. Y. K., Yeung K. S., Fung J. L. F., Beetz C., Bauer P., Luk H. M., Lo I. F. M., Lee C. P., Chung B. H. Y., Kan A. S. Y. (2021). Prenatal and postnatal diagnosis of Schuurs-Hoeijmakers syndrome: Case series and review of the literature. American Journal of Medical Genetics, Part A.

[B25-behavsci-16-00250] Stern D., Cho M. T., Chikarmane R., Willaert R., Retterer K., Kendall F., Deardorff M., Hopkins S., Bedoukian E., Slavotinek A., Schrier Vergano S., Spangler B., McDonald M., McConkie-Rosell A., Burton B. K., Kim K. H., Oundjian N., Kronn D., Chandy N., Chung W. K. (2017). Association of the missense variant p.Arg203Trp in *PACS1* as a cause of intellectual disability and seizures. Clinical Genetics.

[B26-behavsci-16-00250] Tenorio-Castaño J., Morte B., Nevado J., Martinez-Glez V., Santos-Simarro F., García-Miñaúr S., Palomares-Bralo M., Pacio-Míguez M., Gómez B., Arias P., Alcochea A., Carrión J., Arias P., Almoguera B., López-Grondona F., Lorda-Sanchez I., Galán-Gómez E., Valenzuela I., Perez M. P. M., Lapunzina P. (2021). Schuurs–Hoeijmakers syndrome (PACS1 neurodevelopmental disorder): Seven novel patients and a review. Genes.

[B27-behavsci-16-00250] Trujillano L., Ayerza-Casas A., Puisac B., Latorre-Pellicer A., Arnedo M., Lucia-Campos C., Gil-Salvador M., Parenti I., Kaiser F. J., Ramos F. J., Trujillano J., Pié J. (2024). Assessment of quality of life using the kidslife scale in individuals with Cornelia de lange syndrome. Cureus.

[B28-behavsci-16-00250] Van Nuland A., Reddy T., Quassem F., Vassalli J. D., Berg A. T. (2021). PACS1-Neurodevelopmental Disorder: Clinical features and trial readiness. Orphanet Journal of Rare Diseases.

[B29-behavsci-16-00250] Varni J., Limbers C. (2008). The PedsQL Multidimensional Fatigue Scale in young adults: Feasibility, reliability and validity in a university student population. Quality of Life Research.

[B30-behavsci-16-00250] Walkowiak D., Domaradzki J. (2025). Perception of psychosocial burden in mothers of children with rare pediatric neurological diseases. Scientific Reports.

[B31-behavsci-16-00250] Wang M., Schalock R. L., Verdugo M. Á., Jenaro C. (2006). Perspectives of fathers and mothers of children in early intervention programmes in assessing family quality of life. Journal of Intellectual Disability Research.

